# Barriers to condom use among women at risk of HIV/AIDS: a qualitative study from Iran

**DOI:** 10.1186/1472-6874-12-13

**Published:** 2012-05-24

**Authors:** Razieh Lotfi, Fahimeh Ramezani Tehrani, Farideh Yaghmaei, Ebrahim Hajizadeh

**Affiliations:** 1 Reproductive Health Department, School of Nursing and Midwifery, Shahid Beheshti University of Medical Sciences, Tehran, Iran; 2 Reproductive Endocrinology Research Center, Research Institute for Endocrine Sciences, Shahid Beheshti University of Medical Sciences, Tehran, Iran; 3 Health Department, School of Nursing and Midwifery, Shahid Beheshti University of Medical Sciences, Tehran, Iran; 4 Biostatistics Department, Tarbiat Modares University, Tehran, Iran

**Keywords:** Sexual behavior, Barriers, Women, HIV, Qualitative research, Condom

## Abstract

**Background:**

The growing trend of women infected with HIV through sexual transmission is alarming. Factors influencing condom use have not yet been fully identified, especially in countries with conservative cultures and backgrounds. The present study aimed to explore the barriers of condom use in Iranian women at risk of HIV.

**Methods:**

Using the grounded theory methodology, participants’ experiences and their perceptions regarding condom were collected during semi structured in depth interviews. Participants were 22 women, aged 21–49 years, considered to be at risk for HIV, due to their own or their partner’s sexual behaviors. Qualitative analysis of the data was conducted manually and was guided by constant comparative analysis.

**Results:**

Two main barriers, personal and socio-environmental emerged from data analysis. Lack of perceived threat, absence of protective motivation, inadequate knowledge, perceived lack of control, negative attitudes towards condom and misperception were the major personal barriers, while unsupportive environments and cultural norms were the common socio-environmental barriers to condom use among these at risk women.

**Conclusions:**

These critical barriers have to be addressed for implementing effective prevention programs against HIV among populations at risk for HIV.

## Background

The estimated HIV prevalence of over five percent among injection drug users of Iran strongly indicates that the country is being threatened by an HIV epidemic. Despite the establishment of the National Harm Reduction program to overcome the growing trend of HIV among this high risk population, grave concerns are emerging regarding, in particular, sexual transmission of HIV. Although HIV prevalence in the general population in Iran is less than 1%*,* the growing prevalence of sexually transmitted diseases and the major routes of transmission of HIV among infected women are the main concerns in HIV prevention program [1].

One of the most effective strategies in preventing HIV is the correct and continuous use of condoms during sexual relations [2]. Promoting condom use and implementing supportive policies in reducing the growing trend of this disease and limiting the process of its spread play very significant roles [3]. Condom use is a complex behavior and the interactions between individual, environmental, social and structural factors influence its use [4,5]. Different cognitive theories such as the theory of reasoned action, planned behavior and social learning, have not always been able to predict condom use precisely in all situations [6-8]. Studies have shown that nearly fifty percent of young men with more than one sexual partner did not use condoms; furthermore only one third of drug injection users used condom in their last sexual act [1], and some indirect evidence indicates a minimal use of protective behaviors among these populations [9,10].

Promotion of suitable protective behavior among the high risk groups requires deep understanding of factors affecting such attitudes [11]. Qualitative research methods are considered more appropriate for investigating and determining the socio-cultural context of complex health behaviors [12]. The present study aimed to explore the barriers of condom use and the related factors from the perspective of women “at risk” of HIV infection.

## Methods

### Participants and data collection

Twenty-five semi structured in-depth interviews were conducted with the 22 “at risk” women between April and February 2010; in 3 cases, the interviews were repeated to confirm responses given by them to the concept and questions discussed during interviews; this was to make sure that their responses were in line with the concept that had emerged based on their responses. Purposive sampling was used and followed with theoretical sampling according to the codes and categories as they emerged to recruit women with consideration of maximum diversity [13]. In this study, 25 potential participants were recruited, but 3 were excluded because one of them was a transgender, and two were aged less than 18 years.

Theoretical sampling was conducted to describe “concepts that have proven theoretical relevance to the evolving theory” [13]. To do this, participants were recruited in this study based on different age groups, level of education, marital status, risky sexual behaviors, and drug use.

The “at risk” population for the present study was defined as women at risk of HIV due to risky behaviors, either their own or their partner’s, who could be injection drug users or have multiple sexual partners (Table 1). To fulfill inclusion criteria, study participants had to be over 18 years of age, not infected by HIV, Farsi speaking, residents of Tehran and to be having sexual relationships. Participants were selected from among those who came to the three Voluntary Counseling and Testing (VCT**)** centers of the Shahid Beheshti University of Medical Sciences and one NGO Drop In Center (DIC).

**Table 1:** Participant demographics

**Variable**	**Number**	**Percentage**
**Age** (years)		
21-29	5	22.7%
30-39	10	45.5%
40-49	7	31.8%
**Education**		
Illiterate	1	4.5%
Primary and Guidance school	12	54.5%
High School & Diploma	7	31.8%
Higher Education	2	9%
**Marital status**		
Single	2	9%
Married	9	41%
Widow	2	9%
Divorced	9	41%
**Number of Children**		
0	4	18.2%
1-2	16	72.8%
3 or more	2	9%
**Generation of income**		
Yes	10	45.5%
No	12	54.5%
**Drug abuse**		
Yes	12	54.5%
No	10	45.5%
**Homeless**		
Yes	10	45.5%
No	12	54.5%

Participants provided written informed consent before the beginning of interviews and signed a consent form before audio taping. The one illiterate participant also signed the form, after the contents were read out by the researcher to the participant. The semi-structured interview guide consisted of open-ended questions to allow respondents to fully explain their own opinions, perceptions, and experiences. Data collection was carried out by the main researcher, and audio taped. These records were then transcribed verbatim and analyzed consecutively. Durations of interviews lasted between 30 to 107 min (on average 77 min).

### Data analysis

Data collection and analysis were done simultaneously according to the grounded theory methodology. Data collected during interviews were analyzed manually using the Corbin and Strauss method (1998), guided by constant comparative analysis. The constant comparison method is a qualitative data analysis strategy that combines “inductive category coding with a simultaneous comparison of all social incidents observed” [14]. As these incidents are constantly compared with previous incidents, new relationships and typologies are discovered. Open and axial coding was applied to the data. During open coding, each transcript was reviewed several times and the data reduced to codes; then codes that were found to be conceptually similar in meaning were grouped into subcategories; in axial coding the aim was to clarify how the subcategories that emerged were related to preliminary categories. Asking questions and making comparisons, were utilized to find the properties of each concept. As new inferences from data were made, literature was reviewed. Gathering and analyzing data continued until theoretical saturation was reached. “Theoretical saturation” is when repetition and redundancy is observed with the data [15], i.e. when subsequent new information tends to confirm the existing classification themes and new discrepant cases stop appearing, the data has reached saturation. In this study, theoretical saturation emerged when coding of the nineteenth participant was completed. In the first stage of the constant comparison method, the researcher begins by assigning incidents (or units) to categories using a “looks right” or “feels right” basis for assignment [14]. As this constant comparison of incidents and categories was being done, the researcher began to notice the full range of types or dimensions within categories and began to write memos about these ideas. Theoretical and methodological memos are documented. The memo writing and diagrams used to help uncover properties of the categories and to facilitate decision making regarding validity of the model.

### Validity

For data validity, prolonged interaction between researchers and participants and the sufficient time devoted to data collection as well as the active participation of the other members of the team, aside from the main researchers during all stages of the project and the participant’s revision to confirm and verify the conformability and credibility of the data, carried out according to the Strauss and Corbin method [13].

For member checking, four randomly selected participants were given a full transcript of their coded interviews with a summary of the emergent themes to determine whether the codes and themes matched their point of view. The participants provided feedback and all confirmed the concepts and themes that were developed as a result of this study by the research team.

In order to assess the reliability of the data, transcripts of the interviews were read by the researchers and coded and following an interval of several days the text was reread and themes were identified and recoded, following which finally both codes were compared in this sequence until a common theme was achieved and reliability of data was confirmed. Aside from the research team members, results were also checked with some experts of qualitative research, who, although they did not participate in the research, confirmed the fitness of the results. In order to ensure the credibility of data, the whole process of research such as collecting and analysis of data, opinions of supervisors and research in the form of systematic documents were utilized and submitted under the supervision of a qualitative researcher in the field of high risk sexual behaviors. In order to increase transferability, the researcher documented the steps followed in the research and it was decided that these be saved for other researchers to use for research in future studies.

### Ethics

This study has been approved by both the scientific research committee and the Ethics Committee of the Shahid Beheshti University of Medical Sciences.

## Results

The mean age of participants was 34 years (age range: 21–49 years old). The characteristics of participants are shown in Table 1.

Overall, two main categories of barriers emerged from data analysis: Personal and socio- environmental barriers to condom use among “at risk women”. Findings are shown in Table 2.

**Table 2:** A summary of findings

**Personal barriers**	**Socio-environmental barriers**
**Perceived lack of control**	**Unsupportive environment**
Low self-esteem	Inadequate education and information
Low self-efficacy and behavioral skills	Lack of support from the partner
	**Financial needs**
	**Cultural norms**
	Gender roles
	Lack of condom acceptance by the general population
**Loss of protection motivation**	
Emotional needs	
Lustful desire	
Substance abuse	
**Lack of threat**	
Trust and loyalty	
Appearance base judgment	
**Misperception**	
False beliefs about HIV transmission	
Inadequate knowledge	

Personal barriers including the following items:

Perceived lack of control

Feelings and perception of inability and incompetence in the use of condom are a result of several underlying factors, viz. low self-esteem, deficient behavioral skills and lack of self-efficacy in condom use.

### ***Low self-esteem***

Participants with low self-esteem have less sexual protective behaviors. In such circumstances, an individual is deprived of her right to choose and therefore considers herself to be worthless. Low self-esteem is completely evident in the context of addiction and prostitution.

*“Whoever does this kind of work (sex work), has already sold, well … her dignity, conscience and salvation. Well, this is not important for her because she anyway dislikes herself more than enough already and anyone who risks such occupations or this type of work, is deprived of her right of choice regarding condom use.”* (29 years old, multiple sexual partners, drug abuser)

*“Drug addicts do not give importance to themselves; a person must, first of all like herself to want to have choices.”* (26 years old, multiple sexual partners, drug abuser)

They commented that this group lacks the confidence required to convince their partner(s) to use condom and hence are more prone to sexually transmitted diseases.

*“Suggest condoms? No! He never listens to what I say and so why should I bother to explain? If a woman wants to do something, she can do it if she has self-confidence. When a husband does not like his wife* e.g.*in my case, when I ask him to come and be with me and I know he will not come because he prefers to be with somebody else, obviously I lose my self-confidence. We even went to a counselor because I was thinking that perhaps, our marital problems stem from me, that maybe I have some defects…”* (42 years old, husband has multiple partners)

### ***Low self-efficacy and lack of behavioral skills***

This category is based on 2 concepts viz. lack of communication skills in coming to an agreement making a compromise regarding condom use and the lack of self-efficacy in using condom.

*“What I mean is all those people like me, having difficulty with saying “no”, having difficulty in “giving no as an answer”, in the sense that we cannot be what we wanted to be, having difficulty in saying these even 2 words. Not solving the first problem (having sex without the condom) eventually leads to other problems in their life; in the sense that can I by just saying the word “no”, prevent any further difficulties from occurring; the issue has not been resolved, I was not able to convince him and then the problems keep increasing”* (38 year old, multiple sexual partners, drug abuser)

*“Because this man, my husband, who had been in prison and I did not know whether he is sick or not, and he asked me to on the first night after coming home to be with him I, the wife, could not refuse him. Most men in such circumstances will not agree to use of a condom …or things like this.”* (42 years old, husband multi partner)

Not being able to imagine themselves as using condoms in addition to some psychological characteristics (low self-esteem) mentioned earlier, women are all influenced and forced by the power of men’s decisions, financial needs and homelessness.

*“It depends on the common sense of the partner, but when he does not understand, then, there is nothing one can do. Like for example, I want to complain about a relationship without condom, even if I cry, nothing changes-this is the way it is, he does it whatever he wants-however he wants-his way, whether I like it or not.”*(35 years old, multiple sexual partners, drug abuser)

*“No, nobody cares for a street woman. Whoever sympathizes with a street woman? When one is looking for a place to sleep, what can one say? Condom?… he just shrugs …it’s not important to him……”* (29 years old, multiple sexual partners, history of drug abuse)

### Loss of protection motivation

The predominance of some needs, such as emotional needs, lustful desires and also substance abuse were the contributing factors identified for the participants’ lack of motivation to protect themselves against HIV and the reason for disregarding condom use.

### ***Emotional needs***

The need for affection and intimacy are other important factors that deter perception of risk and sometimes force women to ignore the risk, which may explain the reason for neglecting to use the condom.

*“Maybe one reason is lack of affection. It’s very hard for someone to be alone in this big world (sigh), with no one to love you and then you experience the love of the opposite sex and then it grows, making you forget about the risk of the disease. Your attitude changes because your subconscious deceives and tricks you into believing his lies.”* (38 years old, multiple sexual partners, drug abuser)

### ***Lustful desire***

In some of the remarks made by the participants, the desire to achieve sexual pleasure creates a barrier against condom use.

*“Sometimes, something like passion or lust overcomes your reasoning. In the heat of the moment, a person doesn’t care about anything………”* (42 years old, multiple sexual partners, drug abuser)

### ***Substance abuse***

Substance abuse and alcohol consumption strongly affect and influence the ability of women “at risk” to judge safety behavior and the choices and decisions that they can make, making them more susceptible to risk.

“Unfortunately*, because addicts need money……… since if there is no money then there are no drugs, and when there are no drugs, there is no enjoyment, and if there is no enjoyment then there is nothing. In your opinion, when someone does not fear crack and other substances, do you think they will fear hepatitis and HIV?”* (40 years old, multiple sexual partners, drug abuser)

*“In my experience, most women like me do not use the condom……whatever the partner says or asks for, I will submit to in order to get money and in order to obtain drugs, even if I have to have sex without the condom.”* (21 years old, multiple sexual partners, under methadone therapy)

In comments given by women, particularly those that have experienced addiction, they blamed their lack of hope for the future as the main culprit.

*“Some addicts are hopeless and they will say “Hey, HIV? So what? This is not an important thing for them. Some of them have even committed suicide. Hey, what is life for? What am I living for? Why am I still alive?”* (35 year old, multiple sexual partners, history of drug abuse)

Although two participants were against this idea that drug users are not aware of the importance of condom use, and believed that those taking crack and cocaine were for sure using condoms; history of sexually transmitted diseases was one reason being discussed*.*

### Lack of threat

Often, despite the presence of high risk behavior and unprotected sex, the subjects were still not aware of the risk involved or they may just be unable to adequately assess the level of the risk involved. Trust and loyalty and judgment based on appearance create a sense of safety and the absence of the feeling of risk for the majority of participants and as a result, the necessity for condom use is not felt.

### ***Trust and loyalty***

For the majority of participants, marriage is one reason to place their trust in their spouse; therefore condoms are only used if the couple plans to have contraception. Based on the participants’ statements, in order to show devotion, love, and have a warm relationship, the terms often used are trust, confidence, loyalty and safety and a deep sense of loyalty to the man must be established and this constitutes the background for sexual intimacy without condom use.

*“Condom? No! Because I have trust in him and with the exception of myself, he has no other relationships….I think so, based on love and respect, I mean this is how I feel.... I have even tested his love for me.”* (49 years old, multiple sexual partners, drug abuser)

Analysis of the statements of the participants represents the formation of trust in both a temporary marriage (sighe’) and a permanent marriage, both of which are based on togetherness and building love and affection between the couple, who believe that the use of condom seems unnecessary.

*“I myself have a temporary marriage; this type of relationship will lead to the development of trust on both sides. We use condom only for preventing pregnancy, and we must have a lot of trust.”* (25 years old, in a temporary marriage)

Based on the views of other participants, temporary marriage, considering the very essence of its very nature of being temporary can cause, to a very large extent, and in a short time, a feeling of trust and lack of perception of possible risks that can threaten the woman, could all be barriers to condom use and an increase in the risk of HIV.

*“Whoever has a temporary marriage for a month or for 2 months, the risk is higher…the partner will say, “well, you’re my wife, so there’s no reason for using condom.”* (40 years old, multiple sexual partners, drug abuser)

### ***Judgment based on appearance***

For some of the participants, cleanliness, appearance and general health have been established in their minds as being related and individuals who care about cleanliness and hygiene are considered healthy. This leads to decreased perception of risk, and results in abstention from condom use.

*“His body looks healthy because he is not an addict; perhaps my way of thinking is mistaken, but I think he is healthy. Health can be assessed by a person’s color, pale or yellowish….? In my opinion one can tell whether he is healthy or ill,.”* (31 years old, husband injection drug abuser)

### Misperception

#### False beliefs about transmission and prevention of HIV

Some participants in this study pointed out false beliefs regarding infection with HIV and its prevention and treatment as the root cause of the lack of proper knowledge among the population.

*“Because my husband is already infected with this disease, therefore God will not let me have this illness. I think about it this way.”* (30 years old, husband HIV positive)

*“Since he has had a vasectomy maybe I will not get the disease, in the first place we did not have any idea that he has this disease and we had intercourse. I think that if I was supposed to get this disease, I would have been infected in the beginning of our relationship; this is why I believe that since he has had a vasectomy, maybe the disease will not be transferred to me.”* (45 years old, husband HIV positive)

#### Inadequate knowledge

Based on the analysis of the participants’ comments, 3 themes that include inadequate knowledge regarding transmission of HIV, its prevention and its prevalence have been explored. Although all participants had heard about HIV and to some extent know a little about its prevention and transmission, yet, a large number have no knowledge regarding the fields mentioned above and have no idea of how to identify the risk factors; they lack accurate and adequate information.

*“The reason for not using condoms during sexual relationships is that people do not believe or accept that this disease might have such a high prevalence in our country. This is the question I’m asking from you, has it really spread? The people still do not know that the prevalence of HIV might have increased in our country.”* (38 years old, multiple sexual partners, drug abuser)

*“If condom use is not acceptable to the man, well, I will say use anal sex in order that I will be paid more… Risk for HIV?……. Not at all, I think there is no risk since it will be excreted, it will go out and not go upwards, if it’s in the uterus then it will spread upwards into the body. This is what I’ve heard.”* (39 years old, multiple sexual partners, drug abuser)

Socio-environmental barriers including the following items:

Underlying factors contribute to the formation of this theme, concepts such as an unsupportive environment, scarcity of resources and cultural norms.

### Unsupportive environment

Education and insufficient information and a man’s lack of support are the two concepts that form this subcategory.

### ***Inadequate education and information***

According to the participants’ point of view, the lack of protective skills against HIV and the use of condom, stems from the lack of education and lack of awareness on the part of the family particularly the mother, schools and the mass media.

*“I said, well, the mother talks more, she could have talked to me, telling me, this is the way; this is a bottomless pit, but she never talked to me….and where should I learn about it?”* (38 years old, multiple sexual partners)

*“Well, information that should have been given to the people was not; still many do not have any idea how HIV infections are transmitted, or which diseases that are transmitted through sexual contact? Information must be given to the community….in my opinion education must be given in the guidance school; it must be integrated to the school’s curriculum particularly for both boys and girls at the guidance school level (junior high), since this issue is very important.”* (29 years old, multiple sexual partners, history of drug abuse)

### ***Lack of support from the partner***

It has been said many times that the lack of the male’s emotional support for a woman has resulted in the non-acceptance of condom use on the part of the man.

*“Suggestion to use the condom!? No, he never listens to what I say, so why bother explaining? If my spouse liked me, he would accept what I suggest.”... *(42 years old, having a multi partner husband)

### Financial needs

Analysis of the views of the participants suggested that financial needs in the context of homelessness and poverty are important factors to be considered in high risk behaviors.

*“The reason is that…because for money one is forced to do anything, even to the extent of getting HIV.”* (21 years old, multiple sexual partners, drug abuser)

*“Those who live in parks and have no place to go to are helpless and are deprived of the right to refuse or to say the word s “make sure you use the condom”because they are themselves are helpless and resigned. Maybe because they have no place to go or because of financial problems or maybe just because, for some moments, they think ‘I will go and sleep with somebody and give him whatever he wants. No condom? well, it’s not important, even having sex, however he wants it, just so that I can have a bed to lie on and then take a shower, and then be paid my money !”* (21 years old, multiple sexual partners, under methadone therapy)

### Cultural norms

This category includes gender roles and lack of condom acceptance by the general population.

### ***Gender roles***

Most participants in this study have pointed out that repeated inferences such as; men’s freedom of a relationship, double standards, patriarchy and the male’s overpowering strength in a relationship, dependency for the partner in condom use, insecurity in marital relationships and the need of financial support, have been shown to have serious effects in various situations regarding the use of condom for these women to an extent. Participants that had more conservative traditional attitudes toward gender roles had even more perceived barriers of condom use; such an attitude has a negative effect on women’s self-efficacy in protecting themselves from risky and compulsory conditions.

*“If a man has been to prison and when he comes out, he is not aware whether he is infected with HIV or not, and on the first night that the husband returns he has sexual intercourse with his wife (after some months of separation the wife cannot say no) and neither will the husband agree to the use of condom; it’s usually like this…… men will not do such things as use condoms.”* (42 years old, husband multi partner)

*“Sexual relations must be mutual but, really sometimes we are cheated. In some words we are forced to accept it. Sometimes I would say use the condom, and he says ok, or no I want this (sexual relation) and I don’t want that (condom). Sometimes it would happen this way. Men will say if you are not available then we get someone else and have a temporary marriage, but I am a wife and I have no other choice. Men are free to marry ten times….men always have more freedom.”* (34 years old, husband injection drug abuser)

*“Recently, told me several times that he will not allow me to go to the clinic to get condoms, since I usually get condoms from the clinic; he even verbally abused and cursed me. I told him that from now on I will come to you without any condom. What else do you want? Without my knowledge he goes for a laboratory test and he will tell me, never go for a test, that whenever he goes for a test, I will go with him. He himself always goes to get his drugs, and also Hashish laboratory tests, but he will not inform me. After a some days he said “I went to the Red Crescent to have my test” and I reproached him, for not informing so I could also come for a test; I had been with him some days earlier and we did not use a condom, but he all he said was, nothing will happen, don’t worry.”* (29 years old, husband HIV positive)

In traditional gender norms, a woman has no role in her sexual relationships, as a result, it decreases her power in her skills at negotiating condom use.

*“In our society, men are more powerful (with a smile). A man, since he is stronger when he wants sex, it is he who initiates it. He says, it is he who suggested it and that I must agree and say yes! women are more respondents/acceptors rather than initiators; we are embarrassed when we are face to face, which is why we do not use the condom; a woman wants the man to enjoy himself.”* (26 years old, in a temporary marriage)

### ***Lack of condom acceptance by the general population***

Overall there is lack of acceptance of the condom by the general public population.

Nearly all participants believed that almost all of men dislike the condom and hence had negative attitudes towards its use, so they don’t use it regularly.

*“Generally, men dislike condom and usually do not agree to its use; overall its use is not common in Iran.”* (42 years old, multiple sexual partners, drug abuser)

## Discussion

This is the first qualitative study conducted to explore the barriers to condom use in diverse groups of Iranian women at risk for HIV**.** This study demonstrated that individual factors along with socio-environmental factors influenced women’s behavior regarding condom use among such “at risk” populations. The most important barriers to condom use found were low self-esteem, and low self-efficacy in condom use in the context of lack of risk perception especially in legal temporary marriages (Sighe′). Any sense of protective sexual behavior was neglected with drug abuse, financial problems, gender norms and need for emotional dependency and romantic relationships.

Islam attaches far more value to honest behavior. Extramarital sex, homosexuality, alcohol consumption and drug abuse are strictly prohibited in Islam, indicating that Islam consequently always has and still does play a major role in the preventive aspects of the spread of HIV [16,17] and being a Muslim is considered a strong preventive factor for HIV. However, attitudes towards safeguarding numerous legal relationships based on religious laws can be associated with a potential increase in the risk of HIV transmission [9,18]. Therefore one of the most important barriers to condom use in this present study is the perception of trust, commitment and loyalty established by marriage that is in accordance to legal and religious requirements. Hence subjective perception of a safe relationship and the lack of threat perception in a temporary marriage (sighe′), often cause feelings of distrust if condom use is suggested by either spouse; therefore, educational programs aimed at a realistic understanding of susceptibility to risk of HIV must be considered a strategic health priority. Here, the role of religious leaders in the promotion of programs and preventive policies in local and as well as national level has been pointed out and given as an example of such successful programs similar to those conducted in Uganda and Senegal [19]. It seems that the clergy acting as key figures could play an important role in explaining righteous behaviors and the real risk of this disease.

In this study we found that in many cases, having the desire, positive attitude and even the intention to use condoms are not enough to initiate participants to do so and this can be attributed to the fact that sexual behavior is not a single partner decision and that both partners have to participate in this decision making procedure. Being overcome by emotional and sexual excitement, and being unable to prioritize, especially among homeless individuals who need such emotional attachments, hinders their risk perception abilities; despite their knowledge of the high risk of their sexual partner they still trust them, and inevitably neglect using condoms. It seems that different strategies for HIV prevention must be given higher priority, particularly with regards to emotional attachments and to having intimate relationships with high risk women, and to discussions aimed at improving skills of identifying high risk conditions and raising awareness and increasing knowledge about protection as a means of prevention [7,20]. Creating peer groups and social networking that can create a sense of belonging and emotional attachment and social support, and partly fulfill those requirements needs and also assist in making correct decisions, (not decisions based on emotions and excitements) would be of much help in protective sexual behavior [21].

It has been shown that only 29.3% of drug injection users and 7.9% of sex workers of one study in Iran had appropriate information regarding HIV [1]. The present study showed that numerous high risk women had no appropriate knowledge regarding HIV; furthermore their false beliefs regarding its methods of transmission lead to inappropriate behavior. Some of our study participants believed that this disease can only be transmitted through immoral and illegitimate ways and therefore in legal relationships there is no need to use condom as a protective mechanism. The belief of not getting HIV when the man has had a vasectomy is also one example of the false beliefs pointed out in this study. The role of social beliefs has also been stressed in the transmission of HIV [22]. Although information regarding health related behaviors such as prevention of HIV is essential, protective behaviors do not always follow the same sequence [23]. Some of the participants in this study pointed out that despite having some information regarding HIV and its method of transmission, they did not perceive its risk and still refused to regularly use the condom.

In this study, in line with others [24], lack of communication skills such as inability to say no and the lack of negotiation skills regarding its use were highlighted as the main obstacles to condom use. Perceived lack of control in condom use in addition to lack of self-esteem were also mentioned as two main barriers for having sexually protective behavior, in the sense that sexual relations constitute a special mutual relationship and the importance of good communication between couples has been emphasized [25,26]. In addition, the low self-esteem of women who are high risk for HIV has been considered a predictor for high risk behaviors and unprotected sex. The continuous use of condom requires a high level of efficacy [27] however majority of the participants in this study have pointed out that low self-efficacy forces them to usually avoid condom use.

Risk perception is the first step that distinguishes high risk behavior from a more safe behavior [28]. The present study showed that lack of proper risk perception is a common problem among these “at risk” women, which prohibited them from using the condom on a regular basis. Misperception of safety led to improper risk assessment, and a false sense of trust and safety were considered as the main reasons for their not using condoms regularly. Consistent with the findings of other researches [29], in the present study, drug abuse and alcohol are considered to be the two most important factors that decreased risk perception of contracting HIV; this can result from a sense of hopelessness, caused by drug addiction, and the individual’s inability to make timely and appropriate decisions [30]. But a small number of homeless participants mentioned that drug users tend to use condoms more. Ryan and associates have used the theory of alcohol myopia to justify their findings. This theory proposes that alcohol intoxication (and we suggest possibly drug use) restricts attention capacity so that people are highly influenced by the most salient cues in their environment. For sex with casual partners, this may be related to sexual risk [31].

Associations between gender norms, risk of HIV and one’s ability to use condom are issues often documented in literature [32]. Gender norms and interactions that subordinate women in terms of social and economic strength, are recognized as the main barriers of sexual protective behaviors, and they reduce the self-efficacy of women, making them vulnerable to HIV infection [33,34]. In this context the sexual relationship and its protection is a male duty and, as a result, women do not have control over having safe sex [25]. In the present study, gender norms and traditional concepts of sexual relationship weaken women’s negotiation skills; furthermore financial and emotional dependencies, along with cultural obstacles, limit their ability to have safe sex. Women empowerment strategies along with financial and emotional support need to be considered in programs promoting condom usage [35-37]. There exists an association between poverty and a feeling of lack of power in understanding the long term consequences associated with HIV because daily sustaining necessities of life prevent understanding of these consequences [30]. In the present study, the role of poverty in spreading HIV was explicitly expressed. Financial needs and the need for shelter deprived women of the right to choose and ability to decide to use the condom, especially whenever more money suggested for not using condoms. Other studies in assessing the effects of power and poverty and communication skills regarding condom on female sex workers in China have concluded that homeless sex workers use less condoms in comparison to others [36].

As it was stated and represented in the International Conference on Population and Development Action Plan (1994) held in Cairo, to achieve reproductive and sexual health and to combat HIV/AIDS, men’s behavior plays a fundamental role and their cooperation and support in preventing HIV/AIDS can have a positive effect not only on women’s health [38], but also on men’s health as well [39]. Lack of support shown from the husband or sexual partner in the use of condom coupled with the participant’s feeling of inability to make the right choice regarding condom use are the findings of the present study. With the participation of the men and the attendance of couples in the programs for preventive interventions for HIV, we can expect that trust, intimacy and commitment in relationships, encouraging increased communication on condom usage and improvements in communication skills and a supportive environment for safeguarding against HIV will be created [8,40]. Findings of the ten year study in Asia, Africa and Latin America, have shown that men’s participation as well as the mass media have created positive impacts on HIV [41]. Absence of support from the other members of the family particularly the mother and the absence of appropriate communication in the field of sex education, lack of promotion of self-restraint or increased use of condoms in sexual relationships were among the other barriers discussed in our study and other studies as well [42].

### Strengths and limitations

Many of this study’s limitations reflect the limitations of the study setting, where high risk groups such as sex workers are a hidden and stigmatized population. The research team attempted to represent several high risk groups, although the sensitivity of the topic and the fact that these populations are hard to reach may mean that we are neglecting some at-risk populations, especially women who do not refer to VCT and drop in centers. Since there are a limited number of quantitative researches on HIV and women in Iran, and there was not any qualitative research on this topic, the comparability of our results was decreased. In this study, we checked and ensured that all participants were sober by asking a question about her readiness for interview. If someone was not sober, the interview was postponed to a later time.

## Conclusions

We explored the main individual and socio-environmental barriers that influence sexual protective behaviors in Iranian women at risk of HIV, and believe that our findings may help to provide a realistic perspective of the current situation and to the designing of future strategies and implementation of programs for HIV aimed at curbing the increase in its prevalence.

## Competing interests

The authors declare that they have no competing interests.

## Authors’ contributions

All authors contributed to the design of the study. FRT and RL conducted data collection and analysis, and drafted the manuscript. FY and EH assisted with data analysis. All authors read and approved the final paper.

## Pre-publication history

The pre-publication history for this paper can be accessed here:

http://www.biomedcentral.com/1472-6874/12/13/prepub
